# Stable and variable affordances are both automatic and flexible

**DOI:** 10.3389/fnhum.2015.00351

**Published:** 2015-06-19

**Authors:** Anna M. Borghi, Lucia Riggio

**Affiliations:** ^1^Department of Psychology, University of Bologna and Institute of Cognitive Sciences and Technologies, Italian National Research CouncilRome, Italy; ^2^Department of Neuroscience, University of ParmaParma, Italy

**Keywords:** affordances, language comprehension, canonical neurons, mirror neurons, automaticity, grasping, embodied cognition, tools

## Abstract

The mere observation of pictures or words referring to manipulable objects is sufficient to evoke their affordances since objects and their nouns elicit components of appropriate motor programs associated with object interaction. While nobody doubts that objects actually evoke motor information, the degree of automaticity of this activation has been recently disputed. Recent evidence has indeed revealed that affordances activation is flexibly modulated by the task and by the physical and social context. It is therefore crucial to understand whether these results challenge previous evidence showing that motor information is activated independently from the task. The context and the task can indeed act as an early or late filter. We will review recent data consistent with the notion that objects automatically elicit multiple affordances and that top-down processes select among them probably inhibiting motor information that is not consistent with behavior goals. We will therefore argue that automaticity and flexibility of affordances are not in conflict. We will also discuss how language can incorporate affordances showing similarities, but also differences, between the motor information elicited by vision and language. Finally we will show how the distinction between stable and variable affordances can accommodate all these effects.

## Introduction

The study of affordances (Gibson, 1979), i.e., of the invitations to act objects offer to us, is becoming increasingly popular in the last years (for a review, Thill et al., 2013), also due to the increasing spread of embodied and grounded cognition views, according to which there is a strict interaction between perception, action, and cognition. The aim of this paper is to propose a novel view on affordances, which considers the way in which they are represented and vary depending on the context and the task, based on recent evidence obtained in our labs and in other labs.

The paper is organized in two main sections.

In the first section we will claim that different kinds of affordances exist, i.e., stable and variable ones. Both stable and variable affordances are flexible, but to a different extent. We will also consider how these affordances are modulated and constrained by language.

In the second section we will discuss whether affordances are always automatically activated or whether they are contextual dependent. Finally, we will consider some cases in which we might need to “block” affordance activation: the activation of multiple affordances, of broken affordances, and of affordances of dangerous objects.

Overall, we will defend a view according to which automaticity and flexibility are not in conflict.

## Stable and Variable Affordances

The notion of affordance, proposed by Gibson in 1979, has received a lot of interest in the last 15 years. In the ecological perspective adopted by Gibson, affordances consists in the invitation to action offered by the environment to living organisms. Gibson’s theory of affordances is directly related to his overall view of direct perception. According to him, given that our species has evolved in a given ecological niche, the environment directly offers to us the possibility to perceive it correctly, without the mediation of mental representations. Affordances are perceived in a direct way: we do not need to activate objects knowledge to perceive their affordances. Importantly, according to Gisbon affordances are not properties of objects alone but they are relational, since they concern both the organism and the environment, both the subject and the object.

Given that affordances involve both perception and action, it is not surprising that this notion has had a lot of success within embodied and grounded views. However, depending on whether a more or less radical embodied perspective is adopted, this notion has been differently interpreted. While Gibson’s perspective is fully externalist and anti-representational (see also Chemero, 2009), recent authors contributing to the spread of the notion of affordances in psychology and neuroscience have considered affordances as the product of the conjunction, in the brain, of visual and motor experiences (Ellis and Tucker, 2000).

We share this second perspective. This perspective has in our view two important implications. First, it implies that affordances are flexible and continuosly modified and updated thanks to novel experiences. In this respect, there is no discontinuity with Gibson’s view. Second, it ascribes relevance to the interactions between the environment and the organisms as a whole, taking into account not only the dynamics of these interactions but also their neural representation: in this respect, this view departs from Gibson’s externalist view, as a famous quote clarifies: “Ask not what’s inside your head, but what your head’s inside of.” Adopting this second view has led to an increased interest for the neural representation of affordances and has produced impressive behavioral and neural results in the last years.

In order to emphasize both the similarities and the differences between their perspective and Gibson’s view, Ellis and Tucker (2000) have proposed to use the term microaffordance. For research reason, we find the inspiration leading to the proposal of microaffordance highly useful. In fact, in order to understand the processes that are going on during object processing it is very useful to refer to specific action components, as reaching and grasping; the importance of these specific action components is captured in the proposal to use the term micro-affordances (see also Masson et al., 2011). In the following pages we will however stick to the term affordance, since we think it is a good umbrella term, but we will try to formulate a theoretical proposal that takes into account specific action components related to the interaction with specific objects.

We propose (see also Borghi and Riggio, 2009) that, when specific action components are considered, affordances can be distinguished into stable and variable.

We consider stable affordances to derive from rather stable or invariant features or properties of objects, and from their relationship with organisms who interact with them. Imagine for example listening to somebody telling you “Bring here some fruit” or imagine seeing some fruits at a distance. For grasping affordances of fruit the property of size and the grasping action it evokes is rather stable: we may indeed prepare ourselves to interact with cherries using a precision grip, and with apples with a power grip, assuming that these fruits are within our reaching distance. Obviously the size of cherries (or apples) has a certain degree of variability: not all cherries have the same size, but we typically grasp all cherries with a precision rather than with a power grip. The associations between the visual aspects of cherries and apples and the motor response they produce can be incorporated into an object representation, stored in memory (e.g., we “know” that cherries are graspable with a precision grip).

Canonical affordances can be considered as a subset of stable affordances, characterized by a lower degree of stability and higher contextual dependence than purely stable affordances. They derive from properties that vary with respect to the interaction with us, such as orientation, but that can become more stable across multiple experiences. For example, we might consider cherries as having a canonical orientation: they are hardly grasped with the petiole on the lower side, since we typically pick them up from trees, from containers or from plain surfaces, and in all these cases they have the petiole on the upper side. A similar but slightly more complex case is given by cups. The complexity is due to the fact that, differently from natural objects, artifacts typically evoke both manipulative/grasping actions and actions related to object’s use, i.e., they evoke two kinds of actions that do not necessarily coincide (Jax and Buxbaum, 2010). The way we use the term manipulation probably requires some clarification, since this term has been used as well to refer to haptic exploration of objects not guided by a specific goal (Menz et al., 2010). We use this term to refer to the hand posture and grip which are not aimed at using a given object but simply at interacting with it, for example to move it. We propose that canonical affordances are linked to the actions we more typically perform with objects, to the most frequent contexts in which they are embedded and to the most frequent goals with which we approach them. In the case of artifacts these actions, contexts and goals are usually related to their use. Even if we interact with cups in different orientations—for example when we wash them, move them, etc., when we grasp cups to use them, they typically have a specific orientation: they are upright, since we have to hold them to drink the liquid they contain. Due to the higher frequency of this upright orientation when we use cups, it might be useful to store information on cups’ canonical orientation.

Referring to the context can help us to further clarify why canonical affordances can be seen as a subset of stable affordances, characterized by a lower degree of stability and a higher degree of contextual dependency. Stable affordances related to intrinsic properties of objects (Jeannerod et al., 1995), such as those emerging from object size, vary less across contexts and goals. For example, we typically grasp cherries and pencils with a precision grip, independently from the context, for the very simple fact that their dimension affords a precision grip. Canonical affordances, such as canonical orientation, are instead selected by the context. For example, the orientation of a knife for using it in order to cut something and for handling it to somebody else typically differs.

Differently from stable affordances, we consider variable affordances to derive, instead, from rather temporary object characteristics. Furthermore, variable affordances are strictly linked to the actions we are about to perform. Take the following example concerning object location: the location of cherries may vary—they can be on a tree, on a table, and their petiole might be upright but more or less inclined, thus we may need to adapt online our motor responses to the current location of the cherry we intend to grasp. Given the variability of this information, it wouldn’t make sense to store in memory information on it.

Notice that the term “stable” should not be misleading: we do not see any incompatibility between the use of this term and the idea that affordances are processed and responded to online, thus that they might need a certain degree of adjustment of the organism in relationship to objects (see objections by Osiurak, 2013). At the same time, some stable parameters are needed to program actions, in particular if we have to program them offline, without having an object or an entity in front of us. This happens, for example, when someone tells us “Grasp the cherry” or “Lift the hammer” before we have seen or recognized the object we have to interact with. This stability is more the result of a dynamical process than of an *a priori* determination; furthermore, it is not given but it is subject to continuous updating. It could be argued that what we call “stable” affordances are not real affordances but rather consists in simple knowledge of the object. We do not think this is the case, because not only variable but also stable affordances dynamically evoke motor responses. Hence, given that in our view stable and variable affordances are arranged along a continuum, one could speak of “more” stable and “more” variable affordances.

As to their brain localization, some years ago Borghi and Riggio (2009) proposed that stable affordances are represented more ventrally compared to variable ones (see also Young, 2006). In particular, the bipartition of the dorsal stream into a dorso-dorsal and a dorso-ventral system, introduced by Rizzolatti and Matelli (2003), is crucial to capture how these two kinds of affordances are represented in the brain. According to them, the dorso-dorsal stream, corresponding to the dorsal stream as originally defined by Milner and Goodale (Goodale and Milner, 1992; Milner and Goodale, 1995) and contrasted with the ventral stream, is the only stream not related to perception and would be dedicated to the online control of action. The ventro-dorsal stream, instead, would be specifically involved in sensorimotor transformation for grasping, space perception and recognition of actions performed by others (see Gallese, 2007; Binkofski and Buxbaum, 2013; Maranesi et al., 2014). Notice however that these two streams are strictly interconnected and that all three streams—dorso-dorsal, ventro-dorsal and ventral—finish into cortical frontal areas.

A meta-analysis of fMRI studies (Sakreida et al., in preparation) has confirmed that variable affordances are represented more dorsally than stable ones, which are instead represented in bilateral inferior parietal and premotor cortices (dorso-dorsal vs. dorso-ventral stream), even if in the left hemisphere there are overlap areas between the two. This distinction is consistent with clinical observations. Optic ataxia implies indeed impairments during visual reaching of objects, which might be influenced by object orientation (variable affordance). Limb apraxia can be instead characterized by impairments in manipulation of objects, which might be related to object size (stable affordance).

Results obtained with single cell recordings on monkeys and brain imaging data with humans are also informative as to the specific circuits likely involved during processing of specific kinds of affordances (for a review, see Rizzolatti and Craighero, 2004). Data on the monkey’s brain (Raos et al., 2004, 2006) indicate that the visuomotor transformations for grasping objects occur in the anterior intraparietal area (AIP)-F5 circuit, which is devoted to select the most appropriate motor schemas for the actions to be activated. Even if AIP-F5 are better conceived of as a whole, neurons of F5 are more motor and maintain memory of the object also in the dark, while neurons of AIP are more visual and likely render visual affordances available to the motor system. F5 canonical neurons are differently activated depending on the kind of grip objects require (e.g., precision, power) and are not influenced by changing the position of the object in space (Jeannerod et al., 1995). Recent evidence (Bonini et al., 2014) suggests that they are however influenced by the position with respect to the agent’s body, namely they are responsive only when the object is in the peripersonal space. The role played by F5 neurons in motor object representation is consistent with fMRI and PET studies on humans showing that the ventral premotor cortex is activated during observation and imagery of manipulable objects and tools. For example, neuroimaging evidence has shown that images of tools, but not of houses, animals, and faces, activated the ventral premotor cortex (Grafton et al., 1997; Knight et al., 1999; Chao and Martin, 2000), and that the ventral premotor cortex was activated with manipulable objects but not with not manipulable ones (e.g., Gerlach et al., 2002; Kellenbach et al., 2003); the conjunct activation of the left posterior parietal and left premotor cortices can be considered as the human homolog of the canonical neuron system (see Martin, 2007, for a review).

Overall, according to our proposal two ventro-dorsal circuits concern more stable affordances in humans. The first is the phAIP circuit (Orban and Caruana, 2014), which corresponds in humans to the AIP-F5 circuit in monkeys. Both stable and canonical affordances would be represented in this first circuit: it has namely been shown that F5 neurons encode at the single neuron level both the grip and the wrist rotation (Raos et al., 2006). The second circuit, which is present only in humans, would be located in anterior supramarginal gyrus (aSMG), operates in parallel with phAIP and is specifically devoted to tool use (see Orban and Caruana, 2014; on tool use see also Johnson-Frey, 2004; Johnson-Frey et al., 2005).

As to variable affordances, as anticipated we propose they are represented more dorsally, in the dorso-dorsal stream of the dorsal pathway (Rizzolatti and Matelli, 2003). Considering data on the monkey brain, an area candidate to the processing of variable affordances is a third area where grasping neurons are located, namely area F2 (corresponding in humans to the dorsal PM cortex). This area, which receives visual input from the superior parietal lobe and area medial superior temporal (MST), encodes the orientation of the wrist for grasping the object under visual guidance, continuously adjusting online the grip to the object. Notice that the novelty of our proposal does not consist in the anatomical identification of novel neural circuits but rather in connecting these previously identified circuits with the distinction between stable and variable affordances, that hopefully will allow researchers to link behavioral evidence on affordances with such neural underpinnings.

### Similarities and Differences from Other Proposals

In the following section we will discuss the notion of stable and variable affordances in the framework of similar proposals advanced in the literature: the distinction between intrinsic and extrinsic properties, originally proposed by Jeannerod (1981, 1984; Jeannerod et al., 1995), the 2 Action Systems (2AS) proposal (Buxbaum and Kalénine, 2010) and a recent proposal formulated by Orban and Caruana (2014).

Jeannerod (1981, 1984) distinguished between intrinsic object properties, such as size, shape and texture, which are linked to grasping, and extrinsic properties, such as distance and direction, related to object transport in space, which determine the arm and hand position with respect to the object. According to the visuomotor channels hypothesis (Arbib, 1981; Jeannerod, 1981), the visuomotor transformation related to object transportation and to object grasping are namely independent. Different motor schemas would be activated: a circuit pertaining the arm, devoted to transportation, and another specifically focused on the hand, linked to grasping, i.e., to the preshape of the hand while approaching the object and to the enclosure of the hand on the object.

A first difference between the distinction between stable and variable affordances we propose and the distinction between intrinsic and extrinsic object property is that according to our proposal affordances cannot be assimilated to object properties. Affordances are instead relational constructs, i.e., they refer to brain representations of relationships between an organism and one or more objects/entities within a social and physical environment. As said, we agree with Ellis and Tucker (2000) as they consider affordances are patterns of associations, in the brain, of visual and motor experiences.

A second difference is that we do not distinguish between stable affordances related to grasping and variable affordances related to transport. As the examples above should clarify, stable (including canonical) and variable object affordances can emerge both during the transport and the grasping actions.

A further difference concerns the neural underpinnings: the difference between intrinsic and extrinsic properties is indeed linked to the distinction between the grasping and the transportation route (Jeannerod, 1994, 1997; Jeannerod et al., 1995). We propose instead that the distinction between stable and variable affordances is anchored to the difference, within the dorsal route, between the ventro-dorsal and the dorso-dorsal streams (Rizzolatti and Matelli, 2003). The two routes identified by Jeannerod and collaborators as dedicated to transport and to grasping are both represented in the ventro-dorsal route, which in our proposal is dedicated to stable and canonical affordances. Variable affordances would be instead represented in the dorso-dorsal route, mainly used for the online control of actions.

Our view has some similarities with the (2AS) proposal (e.g., Buxbaum and Kalénine, 2010). The main tenet of this proposal is that two different routes to action exist, the Structure and the Function one. The Structure system is bilateral, and it is specialized for visual information related to object shape, size, and location, which is continuously updated. The Function system is left-lateralized, and concerns more stable conceptual knowledge: for example, this system extracts the characteristics of a given action that remains constant over time, such as the typical features characterizing grasping regardless of the specific object to be grasped and of the kind of grip which is used. These systems are not independent but highly interactive and are likely mediated respectively by the dorso-dorsal and by the ventro-dorsal route. According to the 2AS view, artifact objects might evoke at the same time both structural responses and functional ones (see also evidence by Bub et al., 2008), and these actions might interfere. For example, a knife might evoke both manipulation and functional information: a kind of grip adequate to hold it to put it into a drawer (structural response) and another adequate to use it, for example cutting something (functional response). The damage of one of the two systems can lead to impairment: for example, apraxia of tool use would be due to impaired manipulation (structural) knowledge. Jax and Buxbaum (2010) have also demonstrated that the structural and functional systems differ in activation and maintainance time: structural responses are typically quicker than functional ones, but they last less, whereas functional responses are slow and long lasting.

One similarity between our view and the proposal by Buxbaum and Kalenine (see also Binkofski and Buxbaum, 2013), is the assumption that observing a tool allows us to extract information that differs in content and time course, i.e., long term information such as that characterizing stable affordances, and online information such as that characterizing variable affordances. Another similarity is that, in both cases, the dorso-dorsal and the ventro-dorsal routes are the candidate areas for these representations. However, in our view stable affordances are not necessarily dedicated to functional information—for example, for natural objects such as cherries or apples stable affordances can concern the typical way in which we grasp and manipulate them.

Furthermore, while Buxbaum and collaborators stress the potential interference arising between the two action systems, and in particular between manipulation and function, we mainly underline the different temporal and content characteristics of stable and variable affordances. Stable and variable affordances derive indeed from different object characteristics, some of which are more keen to be maintained in long term memory compared to others.

Finally, our proposal has some similarities with the view by Orban and Caruana (2014) who propose that humans have two different parietal circuits: the first, located in phAIP, would be dedicated to grasping and manipulating all kinds of objects; the second, located in the left aSMG, would be specifically devoted to tool use and would be at the basis of technological development in our species. Both would send connections to the vPMC. Importantly, the parallel operation of phAIP and aSMG is a further elaboration of the ventro-dorsal stream (Rizzolatti and Matelli, 2003). According to Orban and Caruana, affordances would refer only to the grasping component, which is related not only to tools but to objects as well, even if the aSMG can contribute in selecting the affordances for phAIP, as demostrated by evidence with patients with ideomotor apraxia. The phAIP component contributes in planning appropriate grasping actions considering objects size and shape, thus corresponding to the canonical neuron systems in monkeys (F5 and AIP), i.e., to the ventro-dorsal stream.

Our proposal is in line with Orban and Caruana’s one since we do believe that it is important to distinguish between motor information related to manipulation and to use, even if this distinction is not at the core of our proposal, and the authors identified two different neural circuits for grasping affordances and tool use in humans. There is clear experimental behavioral and neural evidence supporting the view that grasping and use are different: for example, grasping the handle of an object to use it is disrupted by a semantic task, but not by a visuospatial one (Creem and Proffitt, 2001; see also Creem-Regehr and Lee, 2005). Despite some similarities the focus of our proposal, i.e., the distinction between stable and variable affordances, is obviously different from that of Orban and Caruana. Orban and Caruana limit the use of the term affordances to the activation of object-directed actions that take into account objects’ shape and size. In this sense they adopt a strictly Gibsonian view, according to which accessing to knowledge on the object is not necessary to respond to object affordances. We use the term affordance both to refer to grasping and to use (see Chaigneau et al., 2004, for an integrative view of affordances, intentionality and function in artifacts). The reason why we choose to use the term in both cases is that in humans there are situations in which the distinction is clear, but also many cases in which it is really hard to distinguish between the two. Take for example a fork: to what extent is the proficient use of a fork evoked by characteristics of the fork we process online, to what extent is it due to long-term visuomotor associations between the vision of that particular object and the action of bringing something to the mouth, i.e., to its use? Furthermore, limiting the use of the term affordances to online grasping of objects would not allow us to speak of affordances mediated by language. Given our use of the term affordances, we do not like to claim that affordances are egocentric, as both Orban and Caruana and as Osiurak (2013) seem to do: we report below experimental evidence showing that affordances are modulated by the context, for example that a pen affords different actions when it is presented close to a sheet of paper or to a stapler (Yoon et al., 2010; Borghi et al., 2012; Ellis et al., 2013).

### Stable and Variable Affordances and Language

So far we have illustrated the distinction between stable and variable affordances. One important issue concerns how these affordances are encoded in language (Kaschak and Glenberg, 2000). According to embodied theories of cognition, language is grounded in perception, action and emotional systems (see Gallese and Lakoff, 2005; Barsalou, 2008; Pulvermüller and Fadiga, 2010; Glenberg and Gallese, 2012; Meteyard et al., 2012; see the recent special issues: Borghi and Pecher, 2011; Cappa and Pulvermüller, 2012; Cangelosi and Borghi, 2014). Does this imply that language mirrors exactly the processes and structures of the motor system? We do not believe this is the case. Instead, in line with theories of resuse (Gallese, 2008; Anderson, 2010), we think that language recruits some mechanisms and processes of the perception and motor system, but not necessarily all of them are encoded (see Borghi, 2012, for a more detailed analysis). From this general view the hypothesis follows, that language acts as a sort of filter, encoding only certain kinds of affordances. We will describe some recent studies to clarify our points.

In a behavioral study we asked participants to read a sentence composed by an action vs. an observation verb followed by a noun (e.g., grasp/look at the brush), then they were presented with a photo of an object and had to decide whether the object was the one mentioned in the sentence or not (Borghi and Riggio, 2009). We found that during language processing a motor prototype is formed. This prototype includes stable and canonical affordances, related in this case to object size and canonical orientation: they are indeed encoded in language, while variable affordances are not (see Borghi, 2012, for theoretical development of this issue, and Ferri et al., 2011b; Myachykov et al., 2013, for further evidence). During real interaction with objects the role played by affordances might differ, and in particular the role played by stable affordances might be more marginal, compared to what happens with language.

This hypothesis is supported by evidence by Ferri et al. (2011b). The authors used 3D pictures of objects and asked participants to perform precision or power grips to determine their category (artifact vs. natural object); for artifacts they found a compatibility effect between the grip used to respond and the object size, but only when the objects were presented within the reachable space. In a further experiment participants were required to decide whether the 3D pictures corresponded to previously presented names: in this case the compatibility effect between the grip to respond and the object size was present, but it was not modulated by the space. These data suggest that objects and objects’ names have different motor representations: while objects are characterized both by stable (i.e., shape and size) and variable affordances (i.e., orientation and distance with respect to the perceiver), objects’ names seem to house only stable ones.

Further support to the view that language encoded primarily stable affordances comes from a recent study in which we used mouse tracking to investigate the real-time dynamics of compatibility effects (Flumini et al., 2014). We tracked the time course of a categorization experiment requiring subjects to categorize as natural or artifact pictures of big and small objects. Participants responded using either a big mouse (hand posture compatible with the grasping of big objects) or a small mouse (requiring a precision grip: a hand posture compatible with the grasping of small objects). We found a compatibility effect between the grip required by the mouse and the grip elicited by objects, even if it was irrelevant to the task. A further experiment in which images were substituted by words failed to reproduce the effects. The use of words in this study (as in the previous one) allows to test three different hypotheses, each leading to different predictions. According to the first, words are represented in an abstract, propositional and amodal way, thus no perceptual or motor effect should be found with words. According to the second hypothesis, derived from a purely embodied view, words are grounded in the sensorimotor system, hence the same compatibility effect found with images should be found with words. According to the third hypothesis, derived from theories of reuse (Gallese, 2008; Pezzulo and Castelfranchi, 2009; Anderson, 2010), words are grounded in perception and action systems, but language processing differs to some extent from processing of objects, hence the results with words should not necessarily mirror those obtained with objects. Apparently at odd with a purely embodied view, we did not find the compatibility effect between the grip elicited by the mouse and the grip evoked by the object with words. However, we found that while using the small mouse, and thus performing a precision grip, the processing of artificial small targets was inhibited and the processing of natural small targets was facilitated. This reveals that during language processing participants activated information on the size of the word referent. When the object was an artifact, it provoked an interference with the mouse they held, otherwise a facilitation. The interference is likely due to the fact that artifact words evoke use programs, which are in conflict with the manipulation posture required by the mouse. Such a conflict between manipulation and use is not present with natural objects. This sensitivity to size with linguistic stimuli is in keeping with our third hypothesis. Indeed, it suggests that, since language is a rather sophisticated ability, word processing might not reflect all the dynamics characterizing processing of their referents, in line with theories of reuse. Furthermore, it confirms that language recruits stable affordances, such as size.

### On Dynamic Aspects of Both Stable and Variable Affordances: Some Responses to Osiurak

In a recent paper, Osiurak (2013) criticizes the proposal of stable and variable affordances and proposes that apraxia is not a matter of affordances. Responding to Osiurak’s objections will allow us to better outline our points, in order to avoid any misunderstanding, concerning some important issues. First, we will clarify that the proposal of stable and variable affordances was initially conceived in the context of studies on language processing, and we will show the consequences of this. Second, we will clarify that we are not inclined to use the terms allocentric and egocentric to refer to affordances. Third, we will address the relationship between apraxia and affordances. Fourth, we will try to differentiate between affordances and action goals. We will discuss these four points below.

1. Osiurak (2013) criticizes our view arguing that no affordances about the canonical manipulation of tools can be stored, due to the dynamic character of action. We agree with Osiurak that affordances are flexible and variable (We address this issue more in depth at point 4). At the same time, however, we believe that not only online information but also previous experience play a major role in affordance representation. The role of previous experience is particularly important when we consider affordances as processed offline, as it happens when they are mediated by words or by images. The proposal of stable and variable affordances was firstly advanced in such a context, while discussing the results found by Borghi and Riggio (2009) (see above), showing that during language comprehension we form a motor prototype encoding stable and canonical affordances. Without encoding some stable information no motor recruitment and linguistic comprehension would be possible. At the same time, however, accepting that some stable information must exist does not imply at all denying the importance of the flexibility and contextual dependence of affordances (Mizelle and Wheaton, 2010).

What happens with novel objects? To respond adequately to their affordances we might rely on the context as support (Pellicano et al., 2011). If the context is novel as well, we would in any case need to rely on previous experiences with objects endowed with similar affordances. Jacquet et al. (2012) clearly showed the role of probabilistic cues related to previous experience, together with that of biomechanical constraints, in predicting interaction with novel objects under conditions of visual uncertainty. Overall, we believe that it is difficult to think of objects, entities and situations which are completely novel, in which current experience cannot be traced back to similar previous experiences.

2. Osiurak (2013) claims that manipulation knowledge and stable affordances would be egocentric, since they specify the relationship between the user and the tool. The problem, he argues, is that patients need to form an allocentric representation to solve mechanical problems. Differently from Osiurak, we are not really keen to use the distinction between egocentric and allocentric representation while referring to affordances (see Osiurak, 2013). Rather, we prefer to see affordances as the product of repeated experiences, with a given object or with objects structurally similar to it. This experience is not necessarily an individual experience, but we can benefit from others’ experience. We might observe other people interacting with an object to “capture” the object’s affordance, or we might even see an object or two objects interacting to simulate interaction with them. In this respect, recent evidence on canonical-mirror neurons can be informative. Canonical neurons, which are thought to be the neural underpinnings of affordances (Murata et al., 1997), and mirror neurons (Gallese et al., 1996) were typically considered as segregated: the first ones fire not only when individuals interact directly with objects but also when they observe manipulable objects and the second ones during observation of others’ actions. Recent evidence challenges this dychotomic view (Bonini et al., 2014), showing that canonical-mirror neurons exist. Interestingly, while canonical neurons do not code 90° rotated objects, canonical-mirror neurons do. Furthermore, canonical neurons fire only when objects are located in the peripersonal space, likely due to the connections between area F5 and area F4 (Fogassi et al., 1996; Matelli et al., 1996). Canonical-mirror neurons seem instead to code object as target for both one’s own and other’s action, thus they are not selective only to objects presented in the peripersonal space (for consistent behavioral evidence, see Costantini et al., 2011a,b). This suggests that they could play a role in predicting others’ actions (for a review see Maranesi et al., 2014). In sum, we do not think that affordances are necessarily egocentric, because we can perceive also affordances for others, as demonstrated by recent evidence. Furthermore, we think that to respond to objects’ affordances we take into account the context and the relationships between objects: for example, a cup affords a different action when located near to a coffeepot (we might need to hold it firmly to pour the coffee), near to a spoon (we might need to hold its handle to turn the spoon) or near to an object we want to hide (we might want to turn it upside down). In all these cases, we need to consider the relationship between the objects in the context.

3. As to the relationship between apraxia and affordances, we think, differently from Osiurak, that the possibility that apraxia depends on difficulty in affordances processing is an interesting research avenue that should be explored. For example, disturbed object use and disturbed pantomime in apraxia (Goldenberg, 2009) can be linked to difficulties in responding properly to affordances. In cases of disturbed object use patients typically grasp the object in a wrong way and use it for a wrong purpose: for example, they may grasp a hammer and move it to and fro over the table. In cases of disturbed pantomime the patients either perform the wrong movement or use a body part as object. For example, a patient with disturbed ability to pantomime, when asked to show how to use a comb will scratch his head. Especially interesting are body part as object errors. Here the patient, when asked to use scissors, will move his fingers as it were scissors instead to show that he/she is holding scissors and using them.The fact that such disturbances in object use do not always lead to impairments in object recognition can be explained by the fact that in our view not only object use, but also object manipulation when directed to use can implicitly activate object knowledge (see also Ellis and Tucker, 2000, for a similar view).

4. Finally, with respect to Osiurak we do more clearly differentiate between affordances (i.e., interactions between hand and object) and action goal.

In line with some influential proposals on action representation, such as those derived from ideomotor views (e.g., Prinz, 1997; Hommel et al., 2001), empirical evidence has shown that during action planning the action goal dominates over the hand grip (e.g., van Elk et al., 2011). According to hierarchical views of action representation (e.g., Grafton and Hamilton, 2007), specific motor programs are selected on the basis of the outcome of the action (see however Bonini et al., 2012). We are totally in line with this view. On similar basis, we agree with Osiurak as he highlights that the context and the goal select affordances in a variable and flexible way. But once this has happened, the selected affordances for a given context might be stable.

To clarify with an example: neural and behavioral studies have shown that the way in which we grasp objects might differ depending on the context/goal, and that we are sensitive to this information when we observe others. Fogassi et al. (2005) have shown in a study on the monkey parietal cortex that motor acts, such as “grasping”, are coded differently depending on the action goal (e.g., “grasping for eating” vs. “grasping for placing”) (see also Iacoboni et al., 2005, for an fMRI study on humans). In a similar vein, recent kinematics evidence by Scorolli et al. (2014) demonstrated that subtle variations in the hand posture can suggest whether an individual vs. a cooperative action will be performed (e.g., grasp a cup of tea to drink/to offer it to someone else). The context and the action goal are therefore not independent from affordances. But some aspects remain rather stable: for example, during both grasping for eating and grasping for placing a cherry we use a precision grip, even if the grip orientation and the action preparation vary depending on the action goal.

In sum: we do not intend to deny the flexible interplay between stable and variable aspects that occurs both when we interact with objects and when we process images or words referring to objects. This interplay might however be different depending on whether affordances are processed online, during direct interaction with objects, or whether they are processed through images and words. In the first case stable affordances might play a more marginal role compared to the second.

In particular, we propose that language understanding is tied to and constrained by object affordances, but that language recruits primarily some kinds of affordances, i.e., stable and canonical affordances rather than variable ones (Borghi and Riggio, 2009; Borghi, 2012). As we have seen, evidence from our labs and other labs clearly supports this view.

## Affordances Automaticity Questioned

As discussed above, the notion of affordances has been object of growing interest in the last years, in particular in the framework of embodied and grounded theories of cognition. A variety of studies have been conducted, the majority of which using compatibility effects. For example, in one of their seminal studies Tucker and Ellis (1998) asked participants to decide by pressing with the two hands two different keys on the keyboard to decide whether objects were upright or reversed. Results showed a compatibility effect between the location of the handle of the object (left, right) and that of the key to press (left, right). The results suggest that handles evoke affordances, even if the task does not require to pay attention to them. Evidence like this has been taken as demonstration that observing objects activate affordances, and that affordances are activated automatically, independently from the task at hand (for similar evidence, see Tucker and Ellis, 2001). However, recent evidence suggests more caution in approaching the issue of whether affordances are automatically activated or not; the issue of automaticity contrasted with top-down processing does not seem to be solved but is rather hotly debated (see Buxbaum and Kalénine, 2010; for a recent critical review, see van Elk et al., 2014). First of all, some recent work (Yu et al., 2014) failed to replicate compatibility effects when participants were not explicitly instructed to imagine picking up the pictured objects. More crucially, recent studies have challenged the view according to which affordances are automatically activated, showing that their activation is modulated by the task and the context (e.g., Girardi et al., 2010).

Some results by Riggio et al. (2008) are useful to understand whether affordance effects can be qualified as automatic. The authors presented participants with pictures of two objects with a handle; one object remained on the screen and the other disappeared. They used a modified version of Tucker and Ellis (1998) task, asking half of the participants to judge whether the object that disappeared and the other half to decide whether the object that remained on the screen were upright or reversed. Since disappearing stimuli are dynamic events capturing attention, the target object could or could not be the event capturing attention. The objects were shown above and below or to the left and to the right of a fixation point in order to dissociate the affordance effect (correspondence between handle left-right orientation and response location) and the Simon effect (correspondence between stimulus and response position). The results showed that, while the Simon effect occurred relative to the event capturing attention, the affordance effect, when evident, was always relative to the target object, irrespective of its attentional capturing properties. This result is in keeping with the view that the affordance effect is the consequence of encoding the pragmatic properties of the target, and rules out the possibility that the effect is generated by the attentional capture of the object (or part of it) *per se*. Moreover, these findings suggest that automatic and controlled processes of visual attention may play a differential role in the occurrence of the affordance and Simon effects. In particular, the affordance effect seems to depend on the selection and processing of the objects that are relevant to the task. This finding raises the issue of the automaticity of the activation of affordances, even if the result is not necessarily incompatible with an initial automatic activation of affordances followed by the selection of the affordances relevant to their current task.

Further recent studies, which we will briefly overview below have shown that affordances activation is not independent from the task and is modulated by the context.

### Automatic but Task and Context Dependent

#### Task

A first series of studies has shown that affordances effects are present only when the task requires deep processing of the objects characteristics: for example, shape categorization typically leads to affordance effects, while color categorization does not. To the best of our knowledge, Tipper et al. (2006) were the first who showed that the affordance effect was modulated by the task, since it was present only when participants were required to categorize handles as to their shape, not to their color. In keeping with this result, Pellicano et al. (2010) used torches and demonstrated that when categorizing them on the basis of color (blue vs. red), a Simon effect (compatibility between the goal-directed tip of the object and the location of the key to press to respond) was found, but no affordance effect was present. The affordance effect, intended as the compatibility between the position of the object handle (left, right) and the location of the key to press (left, right), was present only when participants had to decide whether a given torch was upright or reversed. Crucially, the effect was more marked when the torches were switched on, in line with the idea that participants formed a motor simulation of the action of grasping the handle and holding the torch to illuminate. The absence of the affordance effect with a color judgment task, likely due to the fact that color categorization requires superficial processing, challenges the view according to which affordances are activated automatically, i.e., independently from the task at hand (for a computational model on this, see Simione et al., submitted).

#### Context

A further series of studies has started to emphasize the importance of context in responding to affordances.

##### Single objects: near and far space

Recent studies demonstrated that object affordances are only activated when objects are located in the near (peripersonal) but not in the far (extrapersonal) space. Costantini et al. (2010) showed with 3D pictures of everyday objects (e.g., bottle, cup) that objects evoke actions only when they are presented within the portion of the near space that is reachable by the participants.

Further studies with a similar paradigm investigated whether the modulation of the affordance effects due to their location in the near and far space held also with linguistic stimuli. Costantini et al. (2011a) showed participants with 3D objects located in peripersonal vs. extrapersonal space followed by function, manipulation or observation verbs (e.g., “to drink”, “to grasp”, “to look at”). Participants were required to respond releasing a key and performing a simulated grasp when the verb they read was compatible with the presented object. Responses with both function and manipulation verbs were faster when objects were presented in reachable than in the far space, while no difference between the near and far space was present for observation verbs. Results suggest that, during simulation of an action evoked by manipulation and function verbs, objects affordances are primarily activated when objects appear in the participants’ reachable and operational space. Ambrosini et al. (2012) confirmed and extended the previous finding. They used the same paradigm, but introduced a variation: they distinguished between actual and perceived (explicitly estimated by participants) near and far space. Their results confirmed that responses to verbs related to actions were faster in the near than in the far space, while responses to pointing and observation verbs did not differ. Importantly, responses to function and manipulation verbs were faster in the actual near space compared to the perceived near space. This finding suggests that activation of affordances when objects were followed by action verbs is modulated by objects’ location in space with respect to the body. Importantly, this location is computed online and it is not reflected in explicit representations, as the distance estimations participants were required to provide. It should be noted that these results are quite in agreement with the neural counterpart of affordances computation. As already said the AIP-F5 and ventral intraparietal area (VIP)-F4 circuits are interconnected. In particular the VIP-F4 circuit codes the peripersonal space, that coincides with the motor space for arm reaching, regardless of the eye location, thus explaning why the space modulation is limited to action verbs.

One possible question that might rise is the following: given that according to your proposal language encodes stable and (eventually) canonical affordances, but not variable ones, why did you find that variable affordances as those emerging from object’s location and orientation with respect to our own body influence language processing? Notice that the present study does not make use of solely linguistic stimuli, but of linguistic stimuli in combination with pictures—this is likely the reason why action verbs encoded variable affordances as well (see below).

Even if the effect was present with action verbs, it disappeared when object names were presented. Ferri et al. (2011b) used a similar but sligthly different paradigm, in which participants after presentation of the 3D images had to decide by making a reach-to-power or a reach-to-precision response whether the object was congruent with a previously displayed word. They found a compatibility effect between the response and the grip evoked by the object, but, differently from what happened with objects, the effect was not limited within the reaching space. However an important difference exists between the two studies. Costantini et al. (2011a) and Ambrosini et al. (2012) first presented visual objects and then verbs: as already said, in these cases object perception recruits motor information related to their possible interaction only when objects are presented in the near space, determining a priming effect when verbs expressing such an interaction are then presented. Ferri et al. (2011b), conversely, first presented nouns and then visual objects. Results demonstrated that noun processing recruits motor information too, but in this case the motor recruitment is not limited to objects that are then presented in the near space since noun processing cannot specify variables features such as the distance between the object denoted by the noun and the body. Indeed when a noun is presented the VIP-F4 circuit cannot be activated since a physical object is absent. However the size-grasp motor recruitment is still present even if it is not modulated by the space; stressing, in line with embodied theories, that concepts incorporate motor information.

Overall, the studies presented reveal that: (a) affordance activation is modulated by the distance of the object from the body; (b) that this information is encoded in language only to a certain extent, probably due to the fact that the object location is a variable affordance. Further studies with solely linguistic stimuli are probably necessary to better understand how different kinds of affordances are linguistically encoded.

##### Single objects in scene, or more than one object

For a while the majority of studies on affordances have focused on how we respond to single affordances, and to single objects. It is however important to focus also on objects that might evoke different affordances. In everyday life we are indeed typically exposed to multiple affordances. Many objects typically surround us—for example, I might choose to write with the laptop or with a pen I have on my desk. Even the same object can evoke multiple affordances: for example, different parts of an ice cream might evoke grasping and licking. While grasping and licking can be performed at the same time, sometimes objects evoke conflicting actions: for example, a sofa might invite us to sit but also to jump on it, or the same object can elicit different kind of grips. Studies mostly performed in Laurel Buxbaum’s lab have shown that structural information and functional information may conflict while planning actions with objects. Interestingly, these two kinds of information have a different time course: functional information may last longer generating long-term interference, as information in long term memory, while structural information has a rapid decay (Jax and Buxbaum, 2010). Recent work by Kalénine et al. (2014) demonstrated that, depending on the kind of scene in which it is embedded, the same object can evoke a manipulative or a functional grip. They presented images of “conflict” objects, i.e., objects associated with move (clench posture) vs. use (pinch posture) hand postures, as for example a corkscrew. The objects were displayed within everyday scenes, as a kitchen or an office. The results revealed a compatibility effect between the move scene (e.g., drawer for corkscrew) and the clench posture and a more marked compatibility effect between the use scene (e.g., on a bottle for corkscrew) and the pinch response. This result suggests that the same object can evoke different affordances depending on the context. However, the time-course of the process needs to be explored, since the result is compatible with two possibilities: an automatic activation of all object affordances followed by a selection, triggered by the context, of the affordance relevant for the current context, or an early selection determined by the context.

As we have seen, the same object can evoke different affordances, and the context selects which one to activate. Apart from this, objects might be embedded in contexts where multiple objects are present, hence where multiple affordances are activated. Pezzulo et al. (2010) analyzed how expert and novice climbers memorize multiple affordances, i.e., sequences of holds organized in routes of varying difficulty. They found that climbers simulated ascending the route: thus they represented affordances in context, and this influenced their recall. Aside from this study, the great part of evidence concerns online processing of objects or images rather than recall tasks. To our knowledge the only notable exception are the studies recently conducted by Diane Pecher and collaborators, who investigated the role played by affordances in working memory using interfering paradigm (e.g., Pecher, 2013). The authors failed to find that affordances played a role in working memory. This could be due to the fact that, in order to be activated, affordances linked to memory would need deeper processing compared to the more superficial one required by working memory. This is in line with our view, according to which stable and canonical affordances are encoded in long-term memory, while temporary affordances decay rather soon. Their rapid decay can contribute in explaining why variable affordances require continuous monitoring of the relationship between the hand and the object.

So far we used the term “context” referring only to the physical context (e.g., scenes, presence of other objects, etc.). It is however important to determine the influence on affordance activation of both the physical and the social context. To investigate this, some authors have introduced the presence of social cues, for example of an effector interacting or potentially interacting with the object, or of a more complex social context.

##### Physical and social context

Yoon et al. (2010) have demonstrated affordances effects presenting participants with object pairs. Right-handed participants made faster classification responses to pairs of objects displayed in standard co-locations for right-handed actions compared to when the objects are shown in reflected locations. These effects were more marked when participants’ task consisted in deciding if the two objects are typically used together, rather than if objects typically occur in a given context. The effects, which are stronger when an agent is shown holding the objects, disappear when the objects are not viewed from the first-person perspective and when words are presented rather than objects. The data suggest that: (a) participants are sensitive to whether objects are positioned correctly for their own actions; (b) the position information is coded within an egocentric reference frame; (c) the critical representation involved is visual and not semantic; and (d) the effects are enhanced by a sense of agency. The authors interpret the results within a dual-route framework for action retrieval in which a direct visual route, the dorsal one, is influenced by affordances for action, while the ventral route is not. If we consider the further distinction between the dorso-dorso and ventro-dorsal stream, however, we could hypothesize that the process pertains the dorso-dorsal route.

Borghi et al. (2012) presented images of pairs of objects linked by different kinds of relations. They could be functionally related (e.g., scissors-paper) (functional context), thematically related (e.g., scissors, stapler) (spatial context) or not related (e.g., scissors-bottle). The object to be used was positioned on the right. In $\frac{3}{4}$ of the trials a hand appeared, which could be simply close to the object, or interacting with it either with a functional grip, i.e., grasping the object as to use it, or a manipulative grip. Participants were required to decide by pressing a different key on the keyboard whether the two objects were related or not. The results showed a clear effect of the context. Overall, the functional context was processed faster than the spatial one, consistently with the view that artifacts are represented in terms of their use. Most importantly, the interaction between the hand posture and the context was significant. A compatibility effect between context and grip was found: response times were indeed slower when a manipulative grip was presented in a functional context, and when a functional hand posture was displayed in a Spatial context.

The neural mechanisms underlying the described interaction were further investigated by an EEG study with the same stimuli (Natraj et al., 2013). While both Functional and Manipulative postures in the Functional context activated predominantly an early left parietofrontal circuit, the Manipulative posture alone engaged a late right parietofrontal network. Furthermore, bilateral parietofrontal activation increases with the Spatial context, supporting our previous interpretation that, when no functional use of the object is allowed, the motor system tries to make sense of the scene. These EEG results suggest that, when action affordances are not immediately apparent and hand posture does not support action (Manipulative) as well as when the context does not immediately evoke tool use (Spatial context), bilateral activation is increased.

Overall, the two previously described studies highlight the relevance for affordance activation of both the physical context (relations between objects) and of the social cues allowing to detect the intention of the agent, given by the different hand postures. A number of recent studies focus on affordances in a social context (e.g., Sartori et al., 2009; Ferri et al., 2011a; Ellis et al., 2013). We will here illustrate a recent kinematics study by Scorolli et al. (2014) who investigated the role of the physical and social context more in depth, engaging participants in an interaction with real objects and with a real other person (the experimenter). Real objects were presented, which could be linked, as in the previous studies, by no relation, by Spatial relations (e.g., cup-knife) or by Functional relations. The Functional relations could be of two different kinds, i.e., functional-individual or functional-cooperative. With functional-individual relations the two objects are typically used together to perform an individual action (e.g., I typically put the teabag in my own cup), while with Functional-cooperative relations (e.g., cup-teapot) the two objects are used to perform an action that can typically involve somebody else: for example, I typically pour the tea from the tea-pot in the cup of somebody else. Further manipulation of the social intention of the experimenter were introduced: to move the objects the experimenter used either a functional grip or a manipulative grip (e.g., grasping the cup to drink from it or to put it away), and he could observe or not the other (direct vs. indirect gaze). The participants were submitted to two different conditions: in the give condition they had to move the target object toward the experimenter, while in the get condition they moved it toward themselves. The analysis of the kinematic parameters revealed that, during the give condition, the wrist acceleration peak was reached earlier when the other used a functional posture, and the maximal fingers aperture was reached faster when the objects were linked by functional individual than by functional cooperative relations. In the get condition, during visual contact the maximal fingers aperture increased when the experimenter has executed a manipulative grip, as if the participant felt entitled to take the object. This reveals that participants are highly sensitive to cues that might lead to a social or cooperative action. These cues can be found in the relations between objects as well in the characteristics of others that can be indicative of a social intention, such as the gaze and even the hand posture—participants seem indeed to interpret the direct gaze and manipulative grip as leading to a social action.

### Avoiding to Respond to Affordances

Affordances allow us to respond adequately to objects: objects invite us to perform actions with them, in order to reach our goals. However, there might be cases in which, instead of responding to affordances, we may need to avoid responding to the “invitations” we have received.

We will outline a series of cases, which may differ in intensity and specificity, in which such a situation might occur.

#### Multiple Affordances

We have addressed the issue of multiple affordances in the previous session. As we have seen, the studies focusing on multiple affordances are not many, since most of recent experimental work has focused on the interaction with single objects. There are some studies on affordance effects elicited by different parts of the same object (Riggio et al., 2006; Borghi and Riggio, 2009; Pellicano et al., 2010), as well as studies on objects evoking conflicting actions (e.g., Jax and Buxbaum, 2010; Kalénine et al., 2014). Furthermore, some studies present participants with pairs of objects, the affordances of which can be combined to obtain an aim, as for example scissors and papers (Humphreys et al., 2010; Borghi et al., 2012; Natraj et al., 2013; Scorolli et al., 2014), and studies on multiple objects of the same kind (Pezzulo et al., 2010).

Our overall impression is that research so far has not clarified what happens when multiple affordances are activated. It is currently still unclear, whether we activate all possible affordances, or not. There are some possible scenarios: (a) all affordances are automatically activated, and some of them decay, because they are not selected as they are not relevant to the current context/situation and to the current goals of the observer; (b) all affordances are automatically activated, and some of them are actively inhibited to avoid interference between them, since not relevant to the current context and goal; (c) only the single affordance or the subset of affordances relevant to the current context/situation and to our present goals are activated. In the last case the context and the goals would work as an early filter. Both (a) and (b) are in keeping with the influential neural model of action selection described by Cisek (2007). In Cisek’s model, the different objects activate multiple afforded actions automatically, with a later stage of competition in which only one of these actions is selected to be executed.

#### Broken Affordances

A different case is when objects present affordances but they cannot be used, for example because they are broken. It is possible that, in this case, the mechanism is different, i.e., that the affordance is activated but then actively inhibited. In a recent TMS study Buccino et al. (2009) stimulated the left hemisphere hand motor area of participants who observed everyday objects, centrally presented, with a complete or broken handle, positioned to the right or to the left. Results revealed that the Motor Evoked Potential area was larger when the handle was on the right side of the object, but only when the handle was complete. The absence of a difference between right and left when the handle was broken is compatible with the absence of affordance activation when the pragmatic conditions to perform an action are not met. These data suggest that the handle affordances are not activated or that they are activated and then inhibited when the handle is broken, leading to a reduction of activation in the cortical areas typically involved in performing action when the handle is intact. The possibility to inhibit affordances was, for example, shown by Riggio et al. (2006) using an inhibition of return (IOR) paradigm. They presented first whole objects, in which the distinction between the graspable and the ungraspable parts was clearly defined (for instance, in a knife, although we can distinguish between blade and handle, only the handle is used for grasping the object), and then graspable or ungraspable parts of the objects. Participants had to ignore whole objects and to respond to objects parts. Results showed greater inhibitory effects for graspable than for ungraspable parts, specific for the most appropriate action necessary to grasp a specific object. Therefore results suggest distinct inhibitory effects related to the pragmatic features of objects, possibly activated by the neural substrates responsible for sensorimotor transformations required to act properly on an object. If this is the case, the mechanism active with broken affordances would be rather similar to what happens when processing negative action sentences (Tettamanti et al., 2008): the areas typically involved in action representation are recruited and then actively inhibited. A less probable alternative interpretation of the results is that affordances related to the broken handle are inhibited from the very start, hence not activated at all. This would be a case in which the context works as an early filter. Further studies on the time course of the process are necessary to better understand the mechanisms underlying broken affordances activation.

#### Dangerous Objects

A special case is represented by dangerous objects. As in the case of broken affordances, with dangerous objects it is possible that we activate affordances, and then actively inhibit them, or alternatively that we directly avoid to activate them. We will illustrate and discuss below some recent studies we performed in which we contrasted neutral and dangerous objects.

In a first series of studies we presented images of graspable or of dangerous objects, preceded by a hand (a male hand, a female hand and a robotic grasping-hand; a male and a female static-hand) or by a control object, and asked participants to categorize target-objects into artifacts and natural objects by pressing two different keys on the keyboard. Across different experiments, performed with children and adults, we found that response times with dangerous objects were slower compared to those with neutral objects. Let us call this phenomenon a form of inhibition; we will discuss it later in more details. Interestingly, this inhibition was modulated by the hand prime. In a first study with children, we found that the inhibition effect was more marked when the perceived vulnerability of the hand was higher: female hands induced the strongest inhibition, followed by male hands, while robotic hands elicited the lowest one (Anelli et al., 2012); moreover, analyses indicated an effect of motor resonance: the more children and adults perceived the hand as similar to their own hand, the higher was the inhibition. The results of these studies, however, do not allow us to fully disentangle the effects of affordances from the effects of the prime. In addition, it remains unclear whether the slower reactions times (RTs) associated to dangerous objects are due to a late occurring blocking mechanism or the presence of aversive affordances, i.e., to the fact that dangerous objects are perceived as such from the start.

Further studies with different paradigms were performed to better understand the mechanisms underlying affordance activation, deactivation or not activation, in case of dangerous objects. We used a line bisection task (Anelli et al., 2013b). This paradigm is interesting because it allowed us to observe sensitivity to dangerous stimuli with a task not requiring stimulus response compatibility and where the object stimuli did not need to be processed to perform the task (Ohman et al., 2001); furthermore, compared to the above illustrated studies, the object was presented without a hand in potential interaction with it, thus it was easier to capture the effect of the object on its own. In a first study a line was flanked by neutral graspable and by dangerous objects of similar size (e.g., bulb vs. broken bulb; spoon vs. knife; cat vs. porcupine); we found that adolescents and adults tended to misperceive the line midpoint away from the dangerous objects. To understand whether the result was due to an affordance effect (the tendency to approach the graspable object) or to an avoidance effect (the tendency to refrain from the dangerous one) we asked adults to bisect lines flanked by dangerous and neutral objects matched on graspability (both graspable or ungraspable). The results indicate that graspable dangerous objects evoke aversive affordances characterized by the motor tendency to step back and escape. Time course analyses would be necessary to capture precisely how the process unfolds in time.

In a further study (Anelli et al., 2013a) we presented participants, children and adults, with artifact and natural objects, both neutral and dangerous, and asked them to categorize the objects, either by pressing or by releasing two different keys on the keyboard. The critical manipulation consisted in presenting the objects as moving away or toward the participant. Results were rather straightforward: neutral objects responded to faster when they performed an approaching movement, while dangerous objects when they moved away from the participant. No effect of the response typology was present.

To better understand the time course of the process we presented static images of the objects, which were displayed in different sizes (large-medium-small size), as if closer or further away from participants. In Experiment 2, 1 s passed between the presentation of a first image and the displacement of the second, that could be larger, smaller or of the same size, and that represented the go signal; in Experiment 3 the second image was immediately following the first one. The results can be interpreted relying on the different timing of the two experiments: when participants had time to prepare their response (i.e., 1 s passed before the presentation of the go signal) they responded immediately, faster, to larger objects, the most dangerous ones. When they did not have time to prepare themselves, instead, response times were longer, in particular with larger objects. We interpreted this result as due to a sort of freezing effect (see Eder et al., 2014), which was larger the bigger, hence more dangerous were the stimuli.

Overall, all these studies reveal that we are sensitive to dangerous affordances. We respond faster to them when objects or entities with dangerous affordances move toward us. Similarly, we tend to avoid graspable objects with dangerous affordances, as evidence on line bisection reveals. This evidence is in keeping with studies on approach avoidance effects, which show that we tend to attract positively connoted words and to withdraw from negative ones (Chen and Bargh, 1999; van Dantzig et al., 2008; Freina et al., 2009).

As to response times, across the experiments and populations (children, adolescents, adults) we found that responses to dangerous objects are slower than responses to affordances of neutral objects. Further results in the literature are consistent with our findings. Studies on the emotional Stroop effect have shown a general RTs slowdown with aversive stimuli (e.g., Algom et al., 2004). Algom et al. (2004) have proposed that the threatening character of stimuli determines a generic slowdown of responses.

The longer RTs we found with dangerous stimuli can be explained in terms of the mechanisms highlighted by Caligiore et al. (2013) in their TRoPICAL model (see also Caligiore et al., 2010). The model explains negative compatibility effects occurring when participants are required to respond to target-objects while refraining from responding to distractors. According to the model the dorsal and ventral pathways process information related to both the target-object and the distractor. Caligiore et al. (2013) have shown that the prefrontal cortex (PFC) plays a double role, exerting both an inhibitory and an excitatory control (Munakata et al., 2011). In Caligiore et al. (2013), this inhibitory control allows the model to refrain from executing the actions suggested by the distractors; similarly, since PFC can receive inputs from the emotional circuits, it may allow participants to inhibit the tendency to respond to affordances of dangerous objects.

In terms of time course, the slower responses with dangerous objects could be due to two different processes: (a) A two-stages process: we would perceive objects affordances, and plan our actions as a consequence of this; then we would realize that the objects are dangerous and block the planned responses; (b) A more automatic process: we would immediately perceive aversive affordances as such, and we would inhibit any motor response, adopting a freezing behavior. This outcome would occur in particular when dangerous objects are very close to us and we have no time to prepare an exit strategy.

Our data speak in favor of the second hypothesis. At the same time, they reveal that our responses to objects are highly flexible and dependent on the spatial context (near vs. far space), and on the presentation modality of the stimuli, dynamic vs. static (with dynamic objects no effect of the motor response—key press vs. key release—was found, while with static objects clear differences between the two motor responses were observed).

In sum: we have outlined three cases in which we might activate affordances, and then need to suppress them: the cases of multiple affordances, of broken affordances and of dangerous objects. As to multiple affordances, further evidence is needed to understand whether all affordances are automatically activated or whether only affordances relevant to the current context and situation are selected. What is certain is that an increasing number of studies are showing the importance of context for affordances activation. In the case of broken affordances, some of our results suggest that it is possible that the observer actively inhibits the activated affordances or that the affordances are not activated. As to dangerous objects, our results suggest that we do not activate their affordances and then block them, but that we respond directly to aversive affordances.

## Conclusion

Affordances represent an important aspect of our physical and social environment. Our interaction with the surrounding environment is namely potentiated and constrained by them. It is therefore particularly important to understand the mechanisms underlying their activation.

In the first part of the paper we have proposed that two different kinds of affordances exist: stable and variable affordances. As to their brain representation, these two kinds of affordances activate overlapping areas within the dorsal stream but have also different neural underpinnings, since the first activate mainly the dorso-ventral stream, while the second engage primarily the dorso-dorsal one (Sakreida et al., in preparation). We have seen that our proposal is related to Jeannerod’s distinction between intrinsic and intrinsic properties, and that it is strictly linked, but not correspondent, with the view that there might be affordances dedicated to object manipulation and others to object use. Both variable and stable affordances are flexible, even if to a different extent. When it comes to language, we propose that language incorporates only certain kinds of affordances, i.e., stable and canonical rather than variable ones (see Borghi, 2012, for an extensive discussion of this issue). Current evidence obtained in our and other labs supports this view (Borghi and Riggio, 2009; Ferri et al., 2011b; Myachykov et al., 2013; Flumini et al., 2014).

In the second part of the paper we have shown that recent evidence on contextual dependence of affordances may challenge the idea that they are automatically activated. We have briefly reviewed studies showing that the activation of affordances is modulated by the task (superficial vs. deep processing, as in color vs. shape categorization) and by the physical and social context, i.e., by the distance of objects from the body, by the relations between objects, by the scenes in which they are embedded, by the presence of others and by the intentions of others we infer from their behavior.

As we have seen, the data on conceptual dependence are not incompatible with the view that affordances are automatically activated, provided that the selection of the relevant affordances occurs late.

The two parts of this paper, the first concerning kinds of affordances and the second concerning their automaticity, might seem separate and independent, because focused on different aspects. However, we think they are deeply interconnected. The distinction between stable and variable affordances can indeed provide new ways to think of affordances automaticity, and can help advancing new predictions. It is indeed possible that all affordances are automatically activated, and that a competition among them is differently solved depending on the task and the stimuli. We can hypothesize that, when the task and the stimuli are linguistic, functional information “wins” over manipulation, unless the linguistic context clearly primes manipulation (see Lee et al., 2013, for a study highlighting the role of the linguistic context). Similarly, stable affordandes would “win” over variable ones. When the stimuli are not linguistic but consist of real objects and the task involves interaction with them the advantage of stable over variable affordances would disappear. As far as affordances related to manipulation and function are concerned, instead, the competition will be solved differently depending on the context.

In all cases, further evidence on the time course of these processes is needed. In addition, computational models of these processes would be really helpful in providing a synthetic framework and in refining predictions (for current models, see Bonaiuto and Arbib, 2010; Caligiore et al., 2010).

## Conflict of Interest Statement

The authors declare that the research was conducted in the absence of any commercial or financial relationships that could be construed as a potential conflict of interest.

## References

[B1] Algom D. Chajut E. Lev S. (2004). A rational look at the emotional stroop phenomenon: a generic slowdown, not a stroop effect. J. Exp. Psychol. Gen. 133, 323–338. 10.1037/0096-3445.133.3.323 15355142

[B2] Ambrosini E. Scorolli C. Borghi A. M. Costantini M. (2012). Which body for embodied cognition? Affordance and language within actual and perceived reaching space. Conscious. Cogn. 21, 1551–1557. 10.1016/j.concog.2012.06.010 22832214

[B3] Anderson M. L. (2010). Neural re-use as a fundamental organizational principle of the brain. Behav. Brain Sci. 33, 245–266; discussion 266–313. 10.1017/S0140525x10000853 20964882

[B4] Anelli F. Nicoletti R. Bolzani R. Borghi A. M. (2013a). Keep away from danger: Dangerous objects in dynamic and static situations. Front. Hum. Neurosci. 7:344. 10.3389/fnhum.2013.00344 23847512 PMC3698464

[B5] Anelli F. Nicoletti R. Kalkan S. Sahin E. Borghi A. M. (2012). “Humans and robotics hands grasping danger,” in Proceedings of the International Joint Conference on Neural Networks (IJCNN) (New York, USA: WCCI 2012 IEEE World Congress on Computational Intelligence), 1613–1620.

[B6] Anelli F. Ranzini M. Nicoletti R. Borghi A. M. (2013b). Perceiving object dangerousness: An escape from pain? Exp. Brain Res. 228, 457–466. 10.1007/s00221-013-3577-2 23743714

[B7] Arbib M. A. (1981). “Perceptual structures and distributed motor control,” in Handbook of Physiology–The Nervous System II. Motor Control, ed. Brooks V. B. (Bethesda, MD: American Physiological Society), 1449–1480.

[B8] Barsalou L. W. (2008). Grounded cognition. Annu. Rev. Psychol. 59, 617–645. 10.1146/annurev.psych.59.103006.093639 17705682

[B9] Binkofski F. Buxbaum L. J. (2013). Two action systems in the human brain. Brain Lang. 127, 222–229. 10.1016/j.bandl.2012.07.007 22889467 PMC4311762

[B10] Bonaiuto J. Arbib M. A. (2010). Extending the mirror neuron system model, II: what did I just do? A new role for mirror neurons. Biol. Cybern. 102, 341–359. 10.1007/s00422-010-0371-0 20217428

[B11] Bonini L. Maranesi M. Livi A. Fogassi L. Rizzolatti G. (2014). Space-dependent representation of objects and other’s action in monkey ventral premotor grasping neurons. J. Neurosci. 34, 4108–4119. 10.1523/JNEUROSCI.4187-13.2014 24623789 PMC6705269

[B12] Bonini L. Ugolotti Serventi F. Bruni S. Maranesi M. Bimbi M. Simone L.. (2012). Selectivity for grip type and action goal in macaque inferior parietal and ventral premotor grasping neurons. J. Neurophysiol. 108, 1607–1619. 10.1152/jn.01158.2011 22745465

[B13] Borghi A. M. (2012). “Action language comprehension, affordances and goals,” in Language and Action in Cognitive Neuroscience. Contemporary Topics in Cognitive Neuroscience Series, eds Coello Y. Bartolo A. (London: Psychology Press), 125–143.

[B15] Borghi A. M. Flumini A. Natraj N. Wheaton L. A. (2012). One hand, two objects: emergence of affordance in contexts. Brain Cogn. 80, 64–73. 10.1016/j.bandc.2012.04.007 22634033

[B16] Borghi A. M. Pecher D. (2011). Introduction to the special topic embodied and grounded cognition. Front. Psychol. 2:187. 10.3389/fpsyg.2011.00187 21887149 PMC3156979

[B17] Borghi A. M. Riggio L. (2009). Sentence comprehension and simulation of objects temporary, canonical and stable affordances. Brain Res. 1253, 117–128. 10.1016/j.brainres.2008.11.064 19073156

[B18] Bub D. N. Masson M. E. J. Cree G. S. (2008). Evocation of functional and volumetric gestural knowledge by objects and words. Cognition 106, 27–58. 10.1016/j.cognition.2006.12.010 17239839

[B19] Buccino G. Sato M. Cattaneo L. Rodà F. Riggio L. (2009). Broken affordances, broken objects: a TMS study. Neuropsychologia 47, 3074–3078. 10.1016/j.neuropsychologia.2009.07.003 19615389

[B20] Buxbaum L. J. Kalénine S. (2010). Action knowledge, visuomotor activation and emodiment in the two action systems. Ann. N Y Acad. Sci. 1191, 201–218. 10.1111/j.1749-6632.2010.05447.x 20392282 PMC4311774

[B21] Caligiore D. Borghi A. M. Parisi D. Baldassarre G. (2010). TRoPICALS: a computational embodied neuroscience model of experiments on compatibility effects. Psychol. Rev. 117, 1188–1228. 10.1037/a0020887 21038976

[B22] Caligiore D. Borghi A. M. Parisi D. Ellis R. Cangelosi A. Baldassarre G. (eds) (2013). How affordances associated with a distractor object affect compatibility effects: a study with the computational model TRoPICALS. Psychol. Res. 77, 7–19. 10.1007/s00426-012-0424-1 22327121

[B14] Cangelosi A. Borghi A. M. (eds) (2014). Language and action integration: from humans to cognitive robots. Topics Cogn. Sci. 6, 343–558. 10.1111/tops.12103 24943900

[B23] Cappa S. F. Pulvermüller F. (2012). Cortex special issue: Language and the motor system. Cortex 48, 785–787. 10.1016/j.cortex.2012.04.010 22579224

[B24] Chaigneau S. E. Barsalou L. W. Sloman S. A. (2004). Assessing the causal structure of function. J. Exp. Psychol. Gen. 133, 601–625. 10.1037/0096-3445.133.4.601 15584809

[B25] Chao L. L. Martin A. (2000). Representation of manipulable man-made objects in the dorsal stream. Neuroimage 12, 478–484. 10.1006/nimg.2000.0635 10988041

[B26] Chemero A. (2009). Radical Embodied Cognitive Science. Cambridge, MA: MIT Press.

[B27] Chen M. Bargh J. A. (1999). Consequences of automatic evaluation: immediate behavioral predispositions to approach or avoid the stimulus. Pers. Soc. Psychol. Bull. 25, 215–224. 10.1177/0146167299025002007

[B28] Cisek P. (2007). Cortical mechanisms of action selection: the affordance competition hypothesis. Philos. Trans. R. Soc. Lond. B Biol. Sci. 362, 1585–1599. 10.1098/rstb.2007.2054 17428779 PMC2440773

[B30] Costantini M. Ambrosini E. Tieri G. Sinigaglia C. Committeri G. (2010). Where does an object trigger an action? An investigation about affordances in space. Exp. Brain Res. 207, 95–103. 10.1007/s00221-010-2435-8 20931177

[B29] Costantini M. Ambrosini E. Scorolli C. Borghi A. M. (2011a). When objects are close to me: affordances in the peripersonal space. Psychon. Bull. Rev. 18, 302–308. 10.3758/s13423-011-0054-4 21327375

[B31] Costantini M. Committeri G. Sinigaglia C. (2011b). Ready both to your and to my hands: mapping the action space of others. PLoS One 6:e17923. 10.1371/journal.pone.0017923 21483734 PMC3070696

[B32] Creem S. H. Proffitt D. R. (2001). Grasping objects by their handles: a necessary interaction between cognition and action. J. Exp. Psychol. Hum. Percept. Perform. 27, 218–228. 10.1037//0096-1523.27.1.218 11248935

[B33] Creem-Regehr S. H. Lee J. N. (2005). Neural representations of graspable objects: are tools special? Brain Res. Cogn. Brain Res. 22, 457–469. 10.1016/j.cogbrainres.2004.10.006 15722215

[B34] Eder A. B. Rothermund K. De Houwer J. Hommel B. (2014). Directive and incentive functions of affective action consequences: an ideomotor approach. Psychol. Res. [Epub ahead of print]. 10.1007/s00426-014-0590-4 24962237

[B35] Ellis R. Swabey D. Bridgeman J. May B. Tucker M. Hyne A. (2013). Bodies and other visual objects: the dialectics of reaching toward objects. Psychol. Res. 77, 31–39. 10.1007/s00426-011-0391-y 22101988

[B36] Ellis R. Tucker M. (2000). Micro-affordance: the potentiation of components ofaction by seen objects. Br. J. Psychol. 91, 451–471. 10.1348/000712600161934 11104173

[B37] Ferri F. Campione G. C. Dalla Volta R. Gianelli C. Gentilucci M. (2011a). Social requests and social affordances: how they affect the kinematics of motor sequences during interactions between conspecifics. PLoS One 6:e15855. 10.1371/journal.pone.0015855 21283642 PMC3026044

[B38] Ferri F. Riggio L. Gallese V. Costantini M. (2011b). Objects and their nouns in peripersonal space. Neuropsychologia 49, 3519–3524. 10.1016/j.neuropsychologia.2011.09.001 21925191

[B39] Flumini A. Barca L. Borghi A. M. Pezzulo G. (2014). How do you hold your mouse? Tracking the compatibility effect between hand posture and stimulus size. Psychol. Res. [Epub ahead of print]. 10.1007/s00426-014-0622-0 25349026

[B40] Fogassi L. Ferrari P. F. Gesierich B. Rozzi S. Chersi F. Rizzolatti G. (2005). Parietal lobe: from action organization to intention understanding. Science 308, 662–667. 10.1126/science.1106138 15860620

[B41] Fogassi L. Gallese V. Fadiga L. Luppino G. Matelli M. Rizzolatti G. (1996). Coding of peripersonal space in inferior premotor cortex (area F4). J. Neurophysiol. 76, 141–157. 8836215 10.1152/jn.1996.76.1.141

[B42] Freina L. Baroni G. Borghi A. M. Nicoletti R. (2009). Emotive concept-nouns and motor responses: attraction or repulsion? Mem. Cognit. 37, 493–499. 10.3758/MC.37.4.493 19460955

[B43] Gallese V. (2007). The “Conscious” Dorsal stream: Embodied simulation and its role in space and action conscious awareness. PSYCHE 13/1, PSYCHE, 1–20

[B44] Gallese V. (2008). Mirror neurons and the social nature of language: the neural exploitation hypothesis. Soc. Neurosci. 3, 317–333. 10.1080/17470910701563608 18979384

[B45] Gallese V. Fadiga L. Fogassi L. Rizzolatti G. (1996). Action recognition in the premotor cortex. Brain 119, 593–609. 10.1093/brain/119.2.593 8800951

[B46] Gallese V. Lakoff G. (2005). The Brain’s concepts: the role of the sensory-motor system in reason and language. Cogn. Neuropsychol. 22, 455–479. 10.1080/02643290442000310 21038261

[B47] Gerlach C. Law I. Paulson O. B. (2002). When action turns into words. Activation of motor-based knowledge during categorization of manipulable objects. J. Cogn. Neurosci. 14, 1230–1239. 10.1162/089892902760807221 12495528

[B112] Gibson J. J. (1979). The Ecological Approach to Visual Perception. Boston: Houghton Mifflin.

[B48] Girardi G. Lindemann O. Bekkering H. (2010). Context effects on the processing of action-relevant object features. J. Exp. Psychol. Hum. Percept. Perform. 36, 330–340. 10.1037/a0017180 20364922

[B49] Glenberg A. M. Gallese V. (2012). Action-based language: a theory of language acquisition, comprehension and production. Cortex 48, 905–922. 10.1016/j.cortex.2011.04.010 21601842

[B50] Goldenberg G. (2009). Apraxia and the parietal lobes. Neuropsychologia 47, 1449–1459. 10.1016/j.neuropsychologia.2008.07.014 18692079

[B51] Goodale M. A. Milner A. D. (1992). Separate visual pathways for perception and action. Trends Neurosci. 15, 20–25. 10.1016/0166-2236(92)90344-8 1374953

[B52] Grafton S. T. Fadiga L. Arbib M. A. Rizzolatti G. (1997). Premotor cortex activation during observation and naming of familiar tools. Neuroimage 6, 231–236. 10.1006/nimg.1997.0293 9417966

[B53] Grafton S. T. Hamilton A. F. D. C. (2007). Evidence for a distributed hierarchy of action representation in the brain. Hum. Mov. Sci. 26, 590–616. 10.1016/j.humov.2007.05.009 17706312 PMC2042582

[B54] Hommel B. Müesseler J. Aschersleben G. Prinz W. (2001). The Theory of Event Coding (TEC): a framework for perception and action planning. Behav. Brain Sci. 24, 849–878; discussion 878–937. 10.1017/s0140525x01000103 12239891

[B55] Humphreys G. W. Wulff M. Yoon E. Y. Riddoch M. (2010). Neuropsychological evidence for visual- and motor-based affordance: effects of reference frame and object-hand congruence. J. Exp. Psychol. Learn. Mem. Cogn. 36, 659–670. 10.1037/a0019317 20438264

[B56] Iacoboni M. Molnar-Szakacs I. Gallese V. Buccino G. Mazziotta J. C. Rizzolatti G. (2005). Grasping the intentions of others with one’s own mirror neuron system. PLoS Biol. 3:e79. 10.1371/journal.pbio.0030079 15736981 PMC1044835

[B57] Jacquet P. O. Chambon V. Borghi A. M. Tessari A. (2012). Object affordances tune observers’ prior expectations about tool-use behaviors. PLoS One 7:e39629. 10.1371/journal.pone.0039629 22737249 PMC3380924

[B58] Jax S. A. Buxbaum L. J. (2010). Response interference between functional and structural actions linked to the same familiar object. Cognition 115, 350–355. 10.1016/j.cognition.2010.01.004 20156619 PMC2837086

[B59] Jeannerod M. (1981). Intersegmental coordination during reaching at natural visual objects. Atten. Perform. 9, 153–168.

[B60] Jeannerod M. (1984). The timing of natural prehension movements. J. Mot. Behav. 16, 235–254. 10.1080/00222895.1984.10735319 15151851

[B61] Jeannerod M. (1994). The representing brain: neural correlates of motor intention and imagery. Behav. Brain Sci. 17, 187–202. 10.1017/s0140525x00034026

[B62] Jeannerod M. (1997). The Cognitive Neuroscience of Action. Oxford: Blackwell.

[B63] Jeannerod M. Arbib M. A. Rizzolatti G. Sakata H. (1995). Grasping objects: the cortical mechanisms of visuomotor transformation. Trends Neurosci. 18, 314–320. 10.1016/0166-2236(95)93921-j 7571012

[B64] Johnson-Frey S. H. (2004). The neural bases of complex tool use in humans. Trends Cogn. Sci. 8, 71–78. 10.1016/j.tics.2003.12.002 15588811

[B65] Johnson-Frey S. H. Newman-Norlund R. Grafton S. T. (2005). A distributed left hemisphere network active during planning of everyday tool use skills. Cereb. Cortex 15, 681–695. 10.1093/cercor/bhh169 15342430 PMC1364509

[B66] Kalénine S. Shapiro A. D. Flumini A. Borghi A. M. Buxbaum L. J. (2014). Visual context modulates potentiation of grasp types during semantic object categorization. Psychon. Bull. Rev. 21, 645–651. 10.3758/s13423-013-0536-7 24186270 PMC4008714

[B67] Kaschak M. P. Glenberg A. M. (2000). Constructing meaning: The role of affordances and grammatical constructions in sentence comprehension. J. Mem. Lang. 43, 508–529. 10.1006/jmla.2000.2705

[B68] Kellenbach M. L. Brett M. Patterson K. (2003). Actions speak louder than functions: the importance of manipulability and action in tool representation. J. Cogn. Neurosci. 15, 30–46. 10.1162/089892903321107800 12590841

[B69] Knight R. T. Staines W. R. Swick D. Chao L. L. (1999). Prefrontal cortex regulates inhibition and excitation in distributed neural networks. Acta Psychol. (Amst) 101, 159–178. 10.1016/s0001-6918(99)00004-9 10344184

[B70] Lee C. L. Middleton E. Mirman D. Kalénine S. Buxbaum L. J. (2013). Incidental and context-responsive activation of structure-and function-based action features during object identification. J. Exp. Psychol. Hum. Percept. Perform. 39, 257–270. 10.1037/a0027533 22390294 PMC3371276

[B71] Maranesi M. Bonini L. Fogassi L. (2014). Cortical processing of object affordances for self and others’ action. Front. Psychol. 5:538. 10.3389/fpsyg.2014.00538 24987381 PMC4060298

[B72] Martin A. (2007). The representation of object concepts in the brain. Annu. Rev. Psychol. 58, 25–45. 10.1146/annurev.psych.57.102904.190143 16968210

[B73] Masson M. E. Bub D. N. Breuer A. T. (2011). Priming of reach and grasp actions by handled objects. J. Exp. Psychol. Hum. Percept. Perform. 37, 1470–1484. 10.1037/a0023509 21553988

[B74] Matelli M. Luppino G. Govoni P. Geyer S. (1996). Anatomical and functional subdivisions of inferior area 6 in macaque monkey. Soc. Neurosci. Abstr. 22, 2024–2024.

[B75] Menz M. M. Blangero A. Kunze D. Binkofski F. (2010). Got it! Understanding the concept of a tool. Neuroimage 51, 1438–1444. 10.1016/j.neuroimage.2010.03.050 20347045

[B76] Meteyard L. Cuadrado S. R. Bahrami B. Vigliocco G. (2012). Coming of age: A review of embodiment and the neuroscience of semantics. Cortex 48, 788–804. 10.1016/j.cortex.2010.11.002 21163473

[B77] Milner D. Goodale M. A. (1995). The Visual Brain in Action. Oxford: Oxford University Press.

[B78] Mizelle J. C. Wheaton L. A. (2010). The neuroscience of storing and molding tool action concepts: how “plastic” is grounded cognition. Front. Psychol. 1:195. 10.3389/fpsyg.2010.00195 21833254 PMC3153804

[B79] Munakata Y. Herd S. A. Chatham C. H. Depue B. E. Banich M. T. O’Reilly R. C. (2011). A unified framework for inhibitory control. Trends cogn. sci. 15, 453–459. 10.1016/j.tics.2011.07.011 21889391 PMC3189388

[B80] Murata A. Fadiga L. Fogassi L. Gallese V. Raos V. Rizzolatti G. (1997). Object representation in the ventral premotor cortex (area F5) of themonkey. J. Neurophysiol. 78, 2226–2230. 9325390 10.1152/jn.1997.78.4.2226

[B81] Myachykov A. Ellis R. Cangelosi A. Fischer M. H. (2013). Visual and linguistic cues to graspable objects. Exp. Brain Res. 229, 545–559. 10.1007/s00221-013-3616-z 23820977

[B82] Natraj N. Poole V. Mizelle J. C. Flumini A. Borghi A. M. Wheaton L. (2013). Context and hand posture modulate the neural dynamics of tool-object perception. Neuropsychologia 51, 506–519. 10.1016/j.neuropsychologia.2012.12.003 23261936

[B83] Ohman A. Flykt A. Esteves F. (2001). Emotion drives attention: Detecting the snake in thegrass. J. Exp. Psychol. Gen. 130, 466–478. 10.1037//0096-3445.130.3.466 11561921

[B84] Orban G. A. Caruana F. (2014). The neural basis of human tool use. Front. Psychol. 5:310. 10.3389/fpsyg.2014.00310 24782809 PMC3988392

[B85] Osiurak F. (2013). Apraxia of tool use is not a matter of affordances. Front. Hum. Neurosci. 7:890. 10.3389/fnhum.2013.00890 24391576 PMC3868885

[B86] Pecher D. (2013). No role of motor affordances in visual working memory. J. Exp. Psychol. Learn. Mem. Cogn. 39, 2–13. 10.1037/a0028642 22612171

[B87] Pellicano A. Iani C. Borghi A. M. Rubichi S. Nicoletti R. (2010). Simon-like and functional affordance effects with tools: The effects of object perceptual discrimination and object action state. Q. J. Exp. Psychol. (Hove) 63, 2190–2201. 10.1080/17470218.2010.486903 20589580

[B88] Pellicano A. Thill S. Ziemke T. Binkofski F. (2011). Affordances, adaptive tool use and grounded cognition. Front. Psychol. 2:53. 10.3389/fpsyg.2011.00053 21716579 PMC3110812

[B89] Pezzulo G. Barca L. Bocconi A. L. Borghi A. M. (2010). When affordances climb into your mind: Advantages of motor simulation in a memory task performed by novice and expert rock climbers. Brain Cogn. 73, 68–73. 10.1016/j.bandc.2010.03.002 20381226

[B90] Pezzulo G. Castelfranchi C. (2009). Thinking as the control of imagination: A conceptual framework for Goal-Directed Systems. Psychol. Res. 73, 559–577. 10.1007/s00426-009-0237-z 19347359

[B91] Prinz W. (1997). Perception and action planning. Eur. J. Cogn. Psychol. 9, 129–154. 10.1080/713752551

[B92] Pulvermüller F. Fadiga L. (2010). Active perception: Sensorimotor circuits as a cortical basis for language. Nat. Rev. Neurosci. 11, 351–360. 10.1038/nrn2811 20383203

[B93] Raos V. Umiltá M. A. Gallese V. Fogassi L. (2004). Functional properties ofgrasping-related neurons in the dorsal premotor area F2 of the macaquemonkey. J. Neurophysiol. 92, 1990–2002. 10.1152/jn.00154.2004 15163668

[B94] Raos V. Umiltà M. A. Murata A. Fogassi L. Gallese V. (2006). Functional properties of grasping-related neurons in the ventral premotor area F5 of the macaque monkey. J. Neurophysiol. 95, 709–729. 10.1152/jn.00463.2005 16251265

[B95] Riggio L. Iani C. Gherri E. Benatti F. Rubichi S. Nicoletti R. (2008). The role of attention in the occurrence of the affordance effect. Acta Psychol. (Amst) 127, 449–458. 10.1016/j.actpsy.2007.08.008 17905141

[B96] Riggio L. Patteri I. Oppo A. Buccino G. Umiltà C. (2006). The role of affordances in inhibition of return. Psychon. Bull. Rev. 13, 1085–1090. 10.3758/bf03213930 17484440

[B97] Rizzolatti G. Craighero L. (2004). The mirror neuron system. Annu. Rev. Neurosci. 27, 169–192. 10.1146/annurev.neuro.27.070203.144230 15217330

[B98] Rizzolatti G. Matelli M. (2003). Two different streams form the dorsal visual system: anatomy and functions. Exp. Brain Res. 153, 146–157. 10.1007/s00221-003-1588-0 14610633

[B99] Sartori L. Becchio C. Bara B. G. Castiello U. (2009). Does the intention to communicate affect action kinematics? Conscious. Cogn. 18, 766–772. 10.1016/j.concog.2009.06.004 19632134

[B100] Scorolli C. Miatton M. Wheaton L. Borghi A. M. (2014). I give you a cup, I get a cup: A kinematics study on social intention. Neuropsychologia 57, 196–204. 10.1016/j.neuropsychologia.2014.03.006 24680723

[B101] Tettamanti M. Manenti R. Della Rosa P. A. Falini A. Perani D. Cappa S. F.. (2008). Negation in the brain: modulating action representations. Neuroimage 43, 358–367. 10.1016/j.neuroimage.2008.08.004 18771737

[B102] Thill S. Caligiore D. Borghi A. M. Ziemke T. Baldassarre G. (2013). Theories and computational models of affordance and mirror systems: An integrative review. Neurosci. Biobehav. Rev. 37, 491–521. 10.1016/j.neubiorev.2013.01.012 23333761

[B103] Tipper S. P. Paul M. Hayes A. E. (2006). Vision-for-action: The effects of object property discrimination and action state on affordance compatibility effects. Psychon. Bull. Rev. 13, 493–498. 10.3758/bf03193875 17048736

[B104] Tucker M. Ellis R. (1998). On the relations between seen objects and components of potential actions. J. Exp. Psychol. Hum. Percept. Perform. 24, 830–846. 10.1037//0096-1523.24.3.830 9627419

[B105] Tucker M. Ellis R. (2001). The potentiation of grasp types during visual object categorization. Vis. Cogn. 8, 769–800. 10.1080/13506280042000144

[B106] van Dantzig S. Pecher D. Zwaan R. A. (2008). Approach and avoidance as action effect. Q. J. Exp. Psychol. (Hove) 61, 1298–1306. 10.1080/17470210802027987 19086189

[B107] van Elk M. Paulus M. Pfeiffer C. van Schie H. T. Bekkering H. (2011). Learning to use novel objects: a training study on the acquisition of novel action representations. Conscious. Cogn. 20, 1304–1314. 10.1016/j.concog.2011.03.014 21641236

[B108] van Elk M. van Schie H. T. Bekkering H. (2014). Action semantics: A unifying conceptual framework for the selective use of multimodal and modality-specific object knowledge. Phys. Life Rev. 11, 220–250. 10.1016/j.plrev.2013.11.005 24461373

[B109] Yoon E. Y. Humphreys W. W. Riddoch M. J. (2010). The paired-object affordance effect. J. Exp. Psychol. Hum. Percept. Perform. 36, 812–824. 10.1037/a0017175 20695701

[B110] Young G. (2006). Are different affordances subserved by different neural pathways? Brain Cogn. 62, 134–142. 10.1016/j.bandc.2006.04.002 16730868

[B111] Yu A. B. Abrams R. A. Zacks J. M. (2014). Limits on action priming by pictures of objects. J. Exp. Psychol. Hum. Percept. Perform. 40, 1861–1873. 10.1037/a0037397 25045901 PMC8829959

